# Medication Adherence in Adults with Type 2 Diabetes Mellitus Across Gulf Cooperation Council Countries: A Scoping Review of Determinants, Measurement Approaches, and Intervention Evidence

**DOI:** 10.3390/healthcare14213716

**Published:** 2026-11-02

**Authors:** Ahmad Homoud Al-Hazmi, Bashayer Farhan ALruwaili, Afrah Muteb Alshalan, Haripriya Nesanuru, Ibrahim Atallah Alnahidh, Ashokkumar Thirunavukkarasu, Ghadah Zaal Alruwaili, Hanan Farhan S. Alruwaili

**Affiliations:** 1 Department of Family and Community Medicine, College of Medicine, Jouf University, Sakaka 72388, Saudi Arabia; ahhazmi@ju.edu.sa (A.H.A.-H.); ashokkumar@ju.edu.sa (A.T.); 2 Department of Otolaryngology-Head and Neck Surgery, College of Medicine, Jouf University, Sakaka 72388, Saudi Arabia; amalshalan@ju.edu.sa; 3 Department of Community Medicine, Anna Gowri Medical College and Hospital, Puttur, Tirupathi 517583, Andhra Pradesh, India; haripriyachakravarthy@gmail.com; 4 College of Medicine, Jouf University, Sakaka 72388, Saudi Arabia; ibra.al7rbi@gmail.com; 5 Department of Family Medicine, Almukhatat PHCC, Aljouf Health Cluster, Sakaka 72351, Saudi Arabia; gzalrowaili@moh.gov.sa; 6 Department of Nursing, Neonatal Intensive Care Unit, Northern Borders Health Cluster, Arar 73311, Saudi Arabia; hafaalruwaili@moh.gov.sa

**Keywords:** diabetes care, medication adherence, type 2 diabetes mellitus, determinants, barriers, Gulf countries

## Abstract

Background/Objectives: The prevalence of type 2 diabetes mellitus (T2DM) is a significant public health concern in the Gulf Cooperation Council (GCC) countries. Medication adherence is critical in achieving glycaemic control. This scoping review synthesizes recent evidence on medication adherence among patients with T2DM in GCC countries, focusing on determinants and strategies to enhance adherence. Methods: This registered scoping review was developed based on articles published between January 2020 and July 2026, retrieved through searches in PubMed, Scopus, Web of Science, and the Saudi Digital Library. Data were gathered on study design, setting, adherence measurement tools, reported adherence levels, determinants, and interventions. Results: Of 3457 records screened, 549 reports were assessed for eligibility, and 78 studies met the inclusion criteria. Individual studies reported variable and frequently suboptimal medication adherence among adults with T2DM in GCC countries. These study-specific estimates varied widely according to the clinical setting, population, measurement instrument and adherence threshold. Associated factors were patient-related (knowledge, beliefs, and health literacy), therapy-related (regimen complexity, polypharmacy, and side effects), healthcare system-related (continuity of care, patient–physician communication, and access to medicines), and sociocultural (family support, stigma, and religious practices, including Ramadan fasting). Digital health applications, SMS-based interventions, pharmacist-led interventions, and culturally oriented education programs may offer potential benefits for adherence. Nevertheless, their implementation and evaluation remain inconsistent. Conclusions: The available evidence was geographically and methodologically uneven, with considerable heterogeneity in adherence measurement. Standardized measurement approaches and adequately designed longitudinal and intervention studies are needed to strengthen medication-adherence research and practice across GCC countries.

## 1. Introduction

Diabetes mellitus, a chronic metabolic disease, is not just a health issue but a significant societal concern. It is characterized by elevated blood glucose levels, leading to severe damage to vital organs over time [1,2]. Globally, approximately 589 million adults (20–79 years) are living with diabetes mellitus. This represents 10.5% of the world’s population in this age group [2]. Type 2 diabetes mellitus (T2DM) is the most common type of diabetes in the world, with over 90 percent of all cases, and is the largest contributor to the worldwide diabetes prevalence. It is among the rapidly increasing non-communicable diseases that pose a significant burden on the population’s well-being and healthcare systems [2,3]. This growing burden is mainly driven by lifestyle changes, urbanization, ageing, and an increase in obesity, and thus, T2DM is a real global epidemic [1,3].

The Gulf Cooperation Council (GCC) countries (Saudi Arabia, United Arab Emirates [UAE], Kuwait, Oman, Qatar, and Bahrain) lie at the high end of global T2DM prevalence [4]. Recent estimates indicate that diabetes prevalence rates in these countries are significantly higher than the global average of around 10% [4]. A systematic review found that in Saudi Arabia, T2DM prevalence among adults was about 28% (2016–2022) [5]. Across the six GCC countries, the reported prevalence of diabetes ranges from approximately 8% to 22%, demonstrating a substantial but variable burden throughout the region [4,5,6]. The regional risk factors include high rates of obesity, diets that are westernized, low amounts of physical activity, and genetic predispositions. For example, in the GCC, high rates of overweight/obesity, family history, and physical inactivity are considered risk factors [7,8]. This region currently records the highest prevalence of diabetes worldwide, estimated at 17.6%. It also faces the second-largest projected increase in diabetes cases, with the number of affected individuals expected to nearly double, reaching around 163 million by 2050. Alarmingly, this region has the highest proportion of diabetes-related mortality among people younger than 60 years, with about one in five deaths (21.6%) occurring in the working-age population [6]. Overall, T2DM is highly prevalent in the Gulf, more so than in the rest of the world, and poses a significant burden on healthcare systems.

Patient adherence to prescribed therapy is a key factor in the optimal management of T2DM. Adherence refers to taking medications exactly as directed (correct dose, timing, and frequency) [9,10]. Indeed, both clinical and public health experts focus on how enhancing adherence will be more effective for the health of the population compared to discovering novel therapies [9,11]. Adherence to prescribed treatment plans, including metformin, insulin, or combination therapy, is associated with achieving target HbA1c levels and better glycaemic control [12,13]. Notably, good adherence is linked to improved outcomes; for example, adherent patients were more likely to have good glycaemic control than non-adherent patients [14,15]. Conversely, poor adherence is associated with persistently elevated blood glucose, increased healthcare costs, and greater risks of nephropathy, neuropathy, retinopathy, cardiovascular disease, and increased care cost [16,17,18]. Patients may feel well without medication, particularly in the early stages when T2DM can remain asymptomatic; therefore, paying attention to adherence issues is critical at the global level. To avoid conceptual overlap, this review distinguishes adherence-related factors from clinical outcomes. Factors influencing medication-taking behaviour are discussed separately from outcomes such as HbA1c, glycaemic control, diabetes complications, healthcare costs, and quality of life, which are interpreted as outcomes associated with adherence rather than direct determinants of adherence [9,11,13,14].

Due to the high prevalence of T2DM and the reported adherence challenges, there is a clear need to address this issue in the region [4,19,20]. Despite global guidelines emphasizing adherence, the majority of evidence is primarily based in Europe or North America and may not be directly applicable to this region [18,21]. Recent evidence suggests better adherence in some settings, with self-reported adherence of about 90% in one UAE hospital; however, this finding is based on a single study [22]. To date, there has been no comprehensive synthesis of the evolution of Gulf-based evidence on T2DM medication adherence. In particular, the geographical distribution of studies, differences in adherence measurement approaches, and availability of intervention evidence have not been systematically mapped across GCC countries. The heterogeneity of study designs, clinical settings, adherence measurement approaches, associated factors, and interventions makes a scoping review appropriate for systematically mapping this evidence. Therefore, this scoping review aimed to map the evidence published from January 2020 to July 2026 on medication adherence among adults with T2DM in GCC countries, focusing on reported adherence levels, measurement approaches, associated factors, interventions, and remaining evidence gaps. The review question was as follows: What evidence is available on medication-adherence levels, measurement approaches, associated factors, barriers, and interventions among adults with T2DM across GCC countries, and what research gaps remain?

## 2. Materials and Methods

### 2.1. Review Design and Registration

This scoping review was conducted according to the Joanna Briggs Institute methodology for scoping reviews and reported in accordance with the Preferred Reporting Items for Systematic Reviews and Meta-Analyses Extension for Scoping Reviews (PRISMA-ScR) [23,24]. The review protocol was registered in the Open Science Framework (OSF; DOI: https://doi.org/10.17605/OSF.IO/KM43Q). Please find the PRISMA Extension for Scoping Reviews (PRISMA-ScR): Checklist as a supplementary file attachment (Supplementary Table S1). The literature included was heterogeneous in terms of study design, setting, adherence measurement tools, populations, and intervention approaches. Therefore, a scoping approach was considered appropriate to interpret and integrate findings across determinants, barriers, and adherence-improvement strategies. To improve transparency, we used a structured database search, predefined eligibility criteria, and a study selection flow diagram. However, these elements were intended to document the search and selection process and should not be interpreted as indicating a fully systematic review methodology. Selection bias was minimized by searching multiple databases, applying explicit inclusion and exclusion criteria, removing duplicates, and resolving disagreements through discussion among reviewers.

### 2.2. PCC Framework

The eligibility criteria were developed according to the Population–Concept–Context (PCC) framework recommended for scoping reviews. The population comprised of adults aged 18 years or older with T2DM. The concept included medication-adherence levels, measurement approaches, associated factors, barriers, and interventions intended to improve medication adherence. The context comprised healthcare and community settings in the six GCC countries: Saudi Arabia, the United Arab Emirates, Kuwait, Qatar, Bahrain, and Oman.

### 2.3. Search Strategies

An extensive search was conducted in PubMed, Scopus, Web of Science, and the Saudi Digital Library to identify the latest publications (published between 1 January 2020 and 31 July 2026). This timeframe was selected to ensure relevance to current clinical practice, during which significant changes occurred in diabetes management guidelines, therapeutic options, medication adherence assessment, and healthcare delivery across Gulf countries [25,26,27]. The final search of all four databases was conducted on 7 August 2026. Studies published online or available in press by 31 July 2026 were eligible for inclusion. Combinations of keywords and medical subject headings (MeSH) were created to include type 2 diabetes, diabetes mellitus, medication adherence, drug adherence, and the determinants and predictors of medication adherence, as well as barriers, interventions, and the country name in the GCC. We have used the Boolean operators (AND, OR, NOT) to identify relevant articles. The term “compliance” was retained only as a search synonym to capture studies using alternative terminology, while “medication adherence” was consistently used throughout the manuscript.

### 2.4. Inclusion Criteria

The inclusion criteria were that the studies had to be about adult T2DM patients, have been carried out in one or more GCC countries, mention adherence rates and/or determinants and/or interventions, and be in English peer-reviewed journals. Systematic or narrative reviews were considered only for contextual background/discussion or to identify additional primary studies through reference lists but were not included as primary evidence. Exclusion criteria for the articles were as follows: not peer-reviewed (e.g., commentaries, conference abstracts, and editorials), published before 2020, or limited to a specific type of diabetes, such as type 1 diabetes, gestational diabetes, or diabetes in children. Relevance screening of titles and abstracts, reading of full texts, and reading of reference lists of eligible articles were done to identify new studies, and duplicate articles were filtered out across databases. Two reviewers conducted this process, and in cases of disagreement, a third reviewer was consulted to resolve the discrepancy.

### 2.5. Data Charting and Synthesis

**Figure 1:**
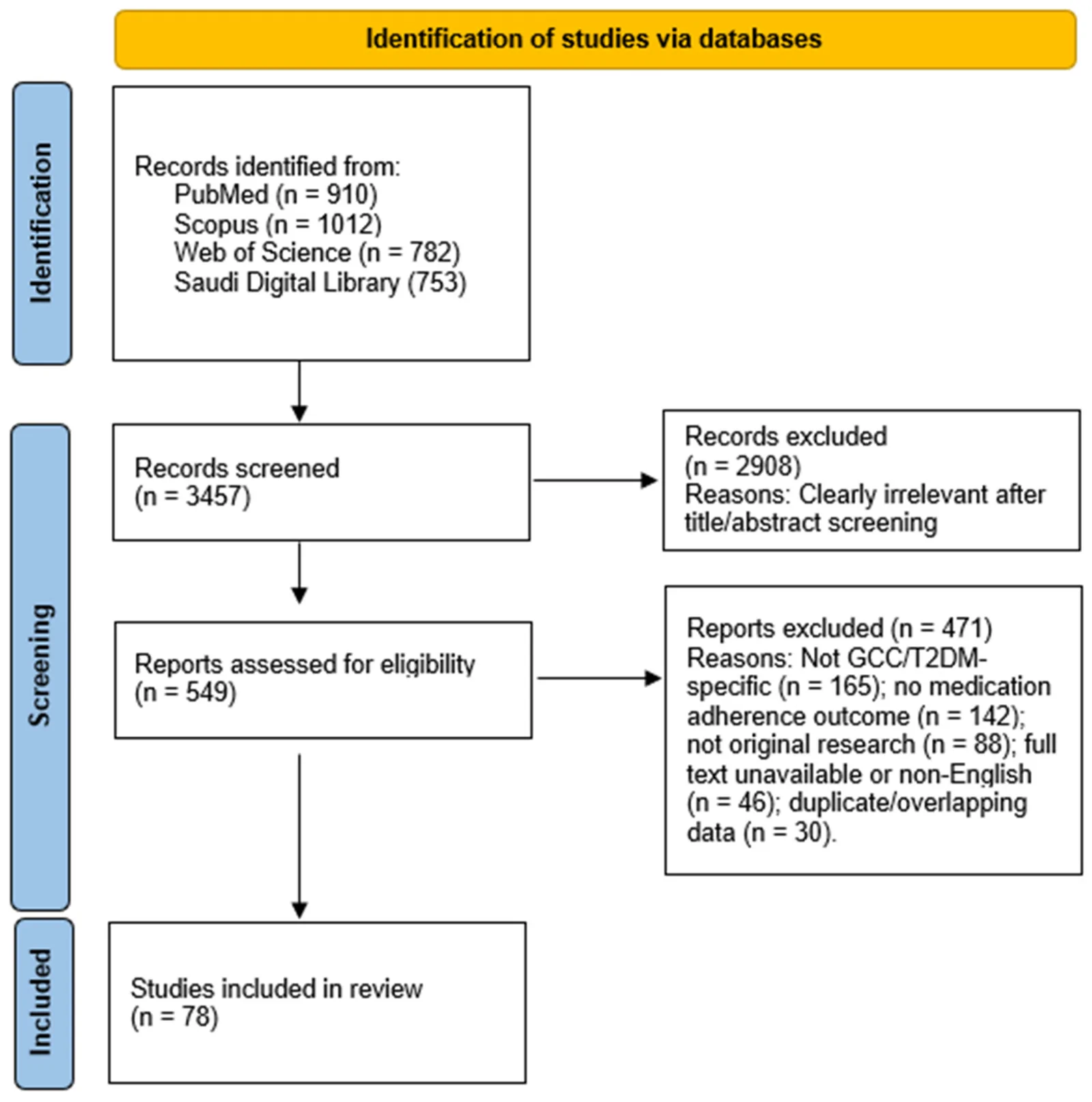
Study selection process flowchart.

Data were charted using a standardized form that captured the author, publication year, country, study design, setting, sample characteristics, adherence measurement instrument, scoring method or cut-off, reported adherence level, associated factors, intervention characteristics, and principal findings. Themes were synthesized across both reported levels of adherence and determinants (patient, therapy, healthcare system, and sociocultural), as well as interventions. Age, education, illness perceptions, complexity of treatment, patient–provider communication, and social support are some of the factors that are addressed in this review as factors related to medication adherence behaviour. Medication adherence was assessed through self-reported measures, medication-taking behaviour, or pharmacy refill-based indicators. Studies reporting glycaemic or digital engagement outcomes were interpreted as providing supplementary clinical or contextual evidence rather than direct measures of medication adherence. Clinical indicators like the HbA1c and glycaemic control are not discussed as a direct associated factor of adherence, but as outcomes of adherence, unless they are analysed as predictors in the original study. For synthesis, findings were distinguished as potential predictors or correlates, reported barriers and facilitators, medication-adherence outcomes, and associated clinical outcomes. Potentially bidirectional associations were identified where appropriate, without inferring causality from cross-sectional evidence. Since most of the studies were cross-sectional, it was assumed that associations were taken rather cautiously, and no causal or bidirectional associations were taken. An overview of search strategies is summarized in Table 1. The final search yielded 910 records from PubMed, 1012 from Scopus, 782 from Web of Science, and 753 from the Saudi Digital Library (3457 records before deduplication). The complete database-specific search strategies, including Boolean operators, field restrictions, and applied filters, are provided in Supplementary Table S2. The number of studies included in the main text is 78, and the selection process is depicted in Figure 1.

## 3. Results

### 3.1. Medication Adherence Levels in GCC

Across the GCC, reported adherence among adults with T2DM varies widely by country, setting (primary vs. specialty care), and instrument (most commonly the eight-item Morisky Medication Adherence Scale [MMAS-8], the General Medication Adherence Scale [GMAS], or the Medication Adherence Rating Scale [MARS]). Adherence estimates should be interpreted cautiously because the included studies used different self-reported tools, such as MMAS-8, GMAS, and MARS, with varying scoring systems, validation contexts, and cut-off definitions. Therefore, adherence rates across countries and settings should be viewed as study-specific indicators rather than directly comparable prevalence estimates. Furthermore, differences in adherence estimates within the same country may reflect variation in study setting, patient profile, treatment complexity, sampling methods, and adherence measurement tools. Recent GCC studies also used objective pharmacy measures (PDC), ARMS-12, MARS-5, SDSCA medication domains, and newly validated phase- or domain-based instruments such as ASMA and the Omani Arabic MAUQ, further limiting direct comparison of adherence estimates [28,29,30,31,32,33,34].

Selected studies conducted in Saudi Arabia reported that approximately half to two-thirds of their participants achieved at least “good” adherence, although estimates differed according to the instruments and adherence thresholds used. Additional Saudi studies demonstrated substantial variation in adherence estimates: high adherence was reported in 7.3% of patients in Majmaah, 25.1% in a Jeddah study of adherence to hypoglycaemic agents, and 51.4% in a pandemic-period survey using the GMAS, while another hospital-based study assessed adherence and its associated factors among patients with T2DM at Najran Armed Forces Hospital [35,36,37,38]. A study from the Eastern Province, using the Saudi-validated GMAS, reported that 62% of patients had good-to-high adherence overall. However, “high” adherence alone accounted for approximately 42%, and higher random blood glucose and insulin-containing regimens were associated with lower scores [39]. A single-centre study in Jazan Province, using the GMAS, classified 66% of 309 adults as adherent, with adherence more likely in older, married, and urban residents, and lower in those with higher HbA1c levels and who were on a greater number of medications [40]. The association between HbA1c and adherence should not be interpreted as evidence of a causal relationship because of the cross-sectional study design.

In Jeddah, a hospital-based study using MMAS reported a mean MMAS-8 of 5.36 ± 1.73 (moderate) and demonstrated the expected gradient between more favourable illness perceptions and higher adherence [41]. A recent study conducted in Saudi Arabia by Alsaidan et al. found that nearly 70% of T2DM patients attending primary health centres had either low or moderate adherence rates to their prescribed medications [42]. Interestingly, a study from Madhina, Saudi Arabia, by Albadrani M et al. in 2025 reported that the majority of their study participants had medium to high levels of medication adherence [11]. Another study by Alanazi M and Alatawi AM from the Tabuk region in Saudi Arabia demonstrated that nearly 23% of their study participants were non-adherent. Additionally, more than one-third of the participants reported complete adherence to their medication regimen and regular attendance at scheduled follow-up visits [43]. Additional Saudi studies found adherence estimates of 68.5% in a primary-care sample and 46.7% in an Eastern Province survey, while another primary-care study classified only 11% as highly adherent [44,45,46]. More recent studies reported high adherence in 48.3% of adults followed by health coaches, ARMS-defined adherence of 32.5% among semaglutide users, and optimal adherence of only 21.6% in a 2026 Jeddah cohort [33,47,48]. In a large objective pharmacy/EHR analysis, PDC-defined adherence was 57.7% for semaglutide and 60.0% for empagliflozin [28].

Two recent Saudi studies further demonstrated variable medication adherence: Alnajjar et al. reported an overall adherence rate of 52.5%, while Elzayat et al. found high adherence in almost half of the participants and partial adherence in nearly one-third [49,50]. Medication-taking behaviour also varied during specific contextual periods: Alshareef et al. documented a significant decline in the mean overall adherence-related score during the COVID-19 lockdown [51], while Mirghani et al. found that full-month Ramadan fasting was negatively associated with medication-adherence scores [52]. Recent intervention studies also reported improved medication adherence following pharmacist-led medication therapy management and structured education combined with telepharmacy follow-up [53,54].

Recent evidence from community and outpatient samples in the UAE indicates inadequate adherence among patients with T2DM. In a UAE cross-sectional MMAS-8 study (*n* = 180 adults), the proportions of low, medium, and high adherence were 61.7%, 28.9%, and 9.4%, respectively, with a significant proportion of suboptimal adherence [19]. A recent study from the UAE by Shaikh SAA et al. found that a considerable proportion of their study participants (90%) demonstrated good medication adherence [22]. In Qatar, estimates vary depending on the sampling frame and disease control status. A hospital-based cross-sectional analysis reported a mean MMAS-8 score of 6.88 ± 1.54, with 57.8% categorized as having high adherence; higher adherence was associated with better self-care and more favourable HbA1c levels. By contrast, a primary-care study of adults with uncontrolled diabetes previously found only 27% adherent, underscoring how clinical context (e.g., unchecked glycaemia) depresses adherence rates. In parallel, Qatar has invested in digital support (e.g., the QDMAT randomised trial of a national diabetes app), though adherence outcomes from that trial are still being accrued [55].

Primary-care data from Muscat shows comparatively favourable adherence. In a 2022 cross-sectional study of 202 adults with T2DM, 65 percent reported high adherence, despite 83 percent experiencing polypharmacy; older age and multimorbidity were common, but the majority rated their health as good/excellent [27]. A multicentre survey of adult patients in Oman with chronic diseases (including diabetes) in 2023 found that adherence was related to better patient–physician relationships and social support, while poorer adherence was associated with increased disease duration and daily dosing frequency, which helps explain adherence variability across Omani contexts [20]. Recent quantitative estimates of T2DM adherence are limited in Kuwait. A 2024 study on information-seeking behaviours among Kuwaiti adults with T2DM noted that patients often rely on family, peers, and social media (e.g., WhatsApp) for disease-management advice—an ecosystem with mixed reliability that may indirectly affect adherence [56]. Up-to-date, T2DM-specific adherence rates are a clear evidence gap. Evidence from Bahrain showed 79.1% adherence overall, but adherence was lower among participants with depression than among those without depression [57]. The multi-country O-SEMA-FAST study, which included Saudi Arabia, the UAE, and Kuwait, found that 68.4% followed oral semaglutide dosing instructions on at least 80% of diary days during Ramadan [58]. A concise country-level comparison of the overall evidence patterns, main associated factors, and distinctive evidence gaps across GCC countries is presented in Table 2.

A detailed comparison of medication-adherence measurement instruments, including scoring approaches, adherence thresholds, validation characteristics, and populations studied, is provided in Supplementary Table S3.

### 3.2. Contextual Factors Influencing Medication Adherence in GCC T2DM Patients

Medication adherence among individuals with T2DM is influenced by a complex interplay of interdependent factors, extending beyond individual patient characteristics. The subsections outline the key domains of factors associated with adherence, patient-related, therapy-related, healthcare system-related, and sociocultural and religious factors, and are based on recent evidence from studies conducted in the GCC region.

#### 3.2.1. Demographic Factors

Age was frequently reported as a factor associated with medication adherence. The scores on adherence showed statistical significance in the Jazan cohort, with individuals above 50 years of age exhibiting higher adherence scores [40]. Similarly, a previous study conducted in eastern Saudi Arabia found a positive correlation between age and adherence [42]. In the study, older patients, who often present with other comorbidities and have experience managing chronic diseases, are more likely to exhibit discipline in population adherence to medication. Marital status also was reported to be significantly associated with adherence among the Jazan community, suggesting that spousal support or extended family setups can be utilized to ensure adherence to prescribed interventions [40]. In a recent Jeddah study, participants aged 51–55 years had lower odds of suboptimal adherence than those aged 40–45 years, while working more than 55 h weekly was also associated with lower odds of suboptimal adherence [48].

There are no significant gender differences in a study from the UAE. Male and female respondents in the Ajman survey showed virtually no difference in the level of adherence to it [59]. Similarly, in an independent study, it was found that there was no significant relationship between gender and overall adherence; however, cost-related adherence was slightly greater in male subjects, which may be explained by the fact that men tend to have more health-related spending. Another study found that female sex was positively associated with medication adherence, whereas age, education, marital status, and occupation were not significant predictors in the adjusted analysis [50]. These findings suggest that gender in the given area has a lesser impact on adherence compared to other demographic factors, including age and marital status [11,40]. In contrast, a primary healthcare study found no significant associations between medication adherence and age, sex, educational level, employment status, or income [47].

A higher level of education and greater disease-specific knowledge are positively associated with better medication adherence [39]. A cross-sectional study in Saudi Arabia assessed diabetes knowledge among patients using the Michigan Diabetes Knowledge Test and adherence using the General Medication Adherence Scale (GMAS) [39]. There was high adherence among around one-third of the participants; however, a weak to moderate positive relationship emerged between knowledge and adherence. The researchers found that patient education on diabetes and diabetic complications is a determinant of adherence that can be altered. A Survey from Madinah found that participants with increased levels of education were more likely to be medium to highly adherent, and they demonstrated the association between low adherence and cultural beliefs, and low socioeconomic status [11].

#### 3.2.2. Psychological and Cognitive Factors Associated with Adherence

Important psychological and cognitive factors associated with adherence include the patient’s perceptions of the disease severity and the perceived need for medication. A recent study on adherence in adults with T2DM in the Middle East and North Africa (MENA region and the GCC as its constituents) has identified key themes: a set of personal factors and perceptions. Specifically, the beliefs about the disease, disease therapies, self-efficacy, and mental health became the cornerstone factors that affected adherence [60]. Non-adherence was correlated with negative beliefs, e.g., the belief that insulin is harmful or that diabetes can be cured. On the contrary, a belief in the chronicity of diabetes and faith in the effectiveness of medications in therapy led to adherence. The emotional reactions of the patients towards the diagnosis also influence the adherence behaviours. A study in Kuwait was a grounded theory study that analysed information-seeking behaviour among adults with T2DM [56]. The participants indicated a sequential process that included denial, acceptance, and adaptation. Others delayed the process of obtaining medical information, sometimes assuming that their symptoms were caused by a temporary condition and thus delaying diagnosis. After diagnosis, the participants experienced confusion and emotional distress, and some of them avoided information intentionally until they thought they were psychologically ready to obtain it. As patients slowly started to accept their illness, they actively pursued viable advice about medications and lifestyle changes and redefined their roles in connection with diabetes-related living.

Qualitative Saudi evidence additionally identified insulin refusal, self-adjustment of doses, beliefs about alternatives, time pressure, forgetfulness, and strained physician relationships as barriers among patients with uncontrolled diabetes [61]. Bahrain data also suggest that depression may be an important psychological correlate of non-adherence [57].

#### 3.2.3. Social and Family Support Networks

Social and family support is an important facilitator of medication adherence. A study conducted in Jazan found that residing in urban areas with increased accessibility to social networks and healthcare resources was associated with higher levels of adherence [40]. A qualitative study has revealed that patients frequently consult with family members, friends, and other patients to re-obtain information and seek reassurance when medical consultations are ineffective [62]. Indicatively, one interviewee told her that she was always pushed by her daughter, who is a student of nutrition, to adhere to her treatment program. Participants also found advice through WhatsApp groups and social media, but they tended to distrust the information and sought validation from their peers [56]. The results demonstrate how family and peer support, as well as community sharing of information, affect adherence behaviours and highlight the need to implement culturally relevant social support interventions.

#### 3.2.4. Religious and Cultural Influences on Medication Practices

Religious and cultural practices may influence medication-taking behaviour during Ramadan in GCC populations [60]. Despite available guidance, knowledge gaps and changes in medication-taking practices during Ramadan remain evident [63,64]. According to a recent report on the self-management assessment, patients delay the initiation of insulin therapy or omit dosages due to existing cultural beliefs and social media messages that discourage medication intake during fasting periods [65]. A study by Mirghani et al. (2023) from Saudi Arabia found that fasting for an entire month (β = −2.90, *p* < 0.001) significantly affects adherence scores, with negative associations indicating lower adherence during fasting periods [52]. A study from Indonesia (a Muslim-majority country) found that adherence significantly declined during Ramadan because of changes in meal times, increased busyness, and a lack of guidance on adjusting medication schedules [66]. O-SEMA-FAST nevertheless showed that more than two-thirds of participants followed fasting-related oral semaglutide dosing instructions on at least 80% of recorded days, indicating that adherence can be supported when the regimen and guidance are adapted to Ramadan [58].

#### 3.2.5. Therapy-Related Determinants

Therapy-related factors include treatment type, dosing complexity, polypharmacy, side effects, and regimen design. Evidence from GCC countries shows that these factors strongly influence medication adherence in T2DM [40,62]. The treatment modality was analysed quantitatively by some authors, and it was found that patients undergoing non-insulin therapies were ranked significantly higher in terms of adherence scores compared to patients prescribed insulin or combination therapies [42]. Oral preparations were also often preferred by the participants due to their convenience, perceived low rate of side effects, and decreased complexity; in contrast, combination therapy was commonly viewed as a sign of a higher level of disease severity. In a survey of T2DM patients in Jazan, the level of adherence was lower among those who received more than one pharmacological agent [40]. In particular, the ingestion of one to three doses daily was associated with better adherence compared to the intake of more than three doses daily. Polypharmacy in T2DM is not unique because of the necessity to simultaneously treat glycaemia, hypertension, and dyslipidaemia [27,40]. The adverse effects present tend to attenuate patients’ desire to continue with treatment. In a national survey of Saudi Arabia, side effects were found to be associated with non-adherence in about 8% of the respondents [49].

Other Saudi studies similarly identified forgetfulness, treatment fatigue, side effects, polypharmacy, and regimen complexity as common barriers [44,45]. Among semaglutide users, lower ARMS adherence was associated with worse BMI and HbA1c, although the cross-sectional design does not establish directionality [33].

#### 3.2.6. Healthcare System and Economic Accessibility Factors

Access gaps, especially in rural areas, are one of the essential factors among patients with unintentional medication non-adherence [65]. Healthcare disruptions may also influence adherence; among adults with T2DM in Jeddah, the mean overall adherence score decreased from 18.49 ± 3.05 before the COVID-19 lockdown to 17.40 ± 3.25 after the lockdown (*p* < 0.001) [51]. Participants reported difficulties accessing medications and healthcare providers, which supports the need for mobile pharmacy outreach. Ease of communication with healthcare providers was also associated with medication adherence; those who found it very easy to ask questions were more likely to be adherent to their medication regimen. Health education sessions and easy-to-book appointments were all associated with better medication adherence [19,20,60]. More recent evidence from Jeddah demonstrated a positive correlation between shared healthcare decision-making and medication adherence, further supporting the involvement of patients in treatment planning and medication-related decisions [50]. Although most GCC states offer free or subsidised drugs to their citizens, expenses are still charged to expatriates who are residents of the country or those with inadequate insurance coverage. One of the studies reported higher adherence in male patients, which was likely because men are the primary source of income and are sometimes able to put priority on medication spending.

On the other hand, economically disadvantaged and women patients might miss doses or make sparse use of medications because of the cost [42]. Saudi participants also noted the problem of cost and the inability to afford medications as one of the factors contributing to low adherence [49]. A 2026 Saudi study further linked suboptimal adherence with unclear treatment plans, inadequate explanation of complications, missed dispensing or appointments, and self-discontinuation [48]. In Makkah primary-care centres, poorer family support, difficulty managing treatment, and side effects were also associated with lower adherence among patients followed by health coaches [47].

#### 3.2.7. Qualitative Evidence on Barriers and Facilitators of Medication Adherence

Qualitative evidence provided additional insights into medication-adherence behaviours in GCC populations. Interview-based studies from Saudi Arabia and Kuwait identified barriers relating to medication beliefs, treatment burden, patient–physician communication, information-seeking behaviours, and social stigma, while family support and access to reliable health information emerged as potential facilitators [56,62]. These findings complement quantitative studies by providing contextual explanations for reported associations between medication adherence, treatment-related factors, and social support.

### 3.3. Interventions and Strategies

Medication adherence in T2DM has been approached from various perspectives, including educational, behavioural, clinical, digital, and psychosocial methods. The intervention evidence was classified according to the outcomes assessed: direct medication-adherence measures (self-reported scales or pharmacy refill-based indicators), clinical outcomes (such as HbA1c, fasting glucose, and weight), or both. Improvements in glycaemic or other clinical outcomes were not considered direct evidence of improved medication adherence unless adherence was measured separately.

Qatar has played a significant role in innovative interventional studies, establishing itself as a pioneer in new trial designs that focus on improving diabetes care. A research study conducted in Qatar in 2020 employed a randomised controlled trial to assess the effectiveness of customized Consumer Medicine Information (CMI) leaflets for patients with T2DM [67]. The traditional pharmacy leaflets often overlook cultural and behavioural determinants; thus, these researchers developed tailor-made CMI handouts that utilized patient-friendly language and did not conflict with the established cultural norms and health beliefs in the area. The results of the trial revealed a positive influence on medication adherence: during a six-month follow-up period, the turnout of participants in the experimental group that became part of the culturally adapted CMI, as well as of the usual care group, had a higher median Medicine Management Adherence Scale (MMAS-8) score than the participants in the usual care group. The authors thus found that medication adherence and possibly glycaemic control in T2DM patients could be improved by using precise and culturally sensitive written information in conjunction with personalized counselling.

Qatar also published a study protocol for the Qatar Diabetes Mobile Application Trial (QDMAT), a randomised controlled trial to evaluate a locally designed mobile application for individuals undergoing insulin therapy [55]. This is an indigenously developed bilingual app featuring glucose tracking, automated alerts, and tools for communicating with educators. The primary outcome of the trial will be the comparative 6-month change in HbA1c levels, and secondary outcomes will include the reduction of missed clinical meetings and an increase in self-management confidence. As QDMAT is a study protocol, its effectiveness in improving medication adherence or glycaemic control has not yet been established by the cited publication.

Saudi Arabia has also prioritized education and pharmacist-based interventions to enhance adherence to T2DM. One of them is the Medication Therapy Management (MTM) program run in the community pharmacy in Riyadh [53]. In this randomised controlled trial, intensive counselling by pharmacists, monthly follow-ups, medication synchronization, and lifestyle advice were provided to patients with uncontrolled T2DM as compared to standard care. The study found that the MTM group achieved clinical benefits. Critically, it was significantly higher in the field of self-reported medication adherence: patients in the pharmacist MTM group were eight times more likely to be adherent than those under usual care. A Saudi project combined health education and telephonic follow-up during the COVID-19 pandemic. This comprehensive program, which included medication education, diet/exercise education, and problem-solving through telepharmacy, showed marked improvements in adherence scores and glycaemic control outcomes in the intervention group compared to the controls [54]. In addition to these studies, diabetes education has become part of the practice in many clinics and hospitals in Saudi Arabia and has been tested with digital reminder systems. For instance, as a practical example, the implementation of the smartphone-based insulin-titration app My Dose Coach™ in Saudi Arabia and Kuwait was associated with a faster achievement of fasting glucose levels, highlighting the potential of app-based aids in insulin-dose adjustments [59]. However, this study primarily reported glycaemic outcomes and application use rather than directly measured medication adherence.

However, a Saudi patient-activation intervention improved activation and several self-care or clinical outcomes without a significant change in the medication-adherence domain, emphasizing that broader self-management gains do not necessarily translate into improved medication taking [32].

The UAE has used telemedicine and electronic health technologies in its medication adherence plans. This study, conducted at the Dubai Diabetes Center, evaluated the effectiveness of telemonitoring in patients with T2DM who had previously been lost to follow-up [68]. This group offered them home-based telehealth services, including digital glucometers, Bluetooth blood-pressure monitors, and smart pill dispensers, and scheduled home-based consultations over a 3-month period. The results were very positive: the average level of glycated haemoglobin (HbA1c) dropped to 7.4% at the end of the intervention, which is a positive change of about 3% in 90 days. Also, 50% of the initially non-adherent group was able to have HbA1c levels under 7% after three months of telemonitored treatment. There was also a decrease in mean fasting plasma glucose by 40 mg/dL and slight weight loss. As this study demonstrates, telemedicine and remote monitoring can be utilized to re-engage patients and may help re-engage patients and improve adherence and metabolic control, even among patients who were previously non-adherent.

A retrospective observational evaluation of the GluCare hybrid model has demonstrated promising clinical outcomes in T2DM. The integration of in-clinic consultations with continuous remote monitoring leads to substantial improvements in glycaemic control and clinical parameters [69]. The case group showed significant HbA1c improvements (–2.19%), especially in higher baseline levels. Controls showed smaller improvements. Higher digital interactions correlated with better outcomes. Patients with ≥11 interactions showed significant reductions in HbA1c (–2.38%) and weight (–6.00 kg). These findings demonstrate potential benefits of technology-supported care for glycaemic control and patient engagement; however, the GluCare study did not report a formal medication-adherence outcome, limiting conclusions regarding its effectiveness in improving adherence.

Saudi observational evidence also found higher adherence among patients exposed to telemedicine than among those receiving in-person care, although causality cannot be inferred [31]. In the UAE, a 12-month pharmacist-led randomised trial improved medication possession ratio compared with usual care, supporting a combined approach involving medication review, counselling, educational material, and a mobile application [70].

The Omani primary healthcare networks frequently conduct special diabetes clinics and follow-ups for non-communicable diseases (NCDs) in all regions. The country quickly expanded remote health services, such as telephone consultations and medication delivery, during the COVID-19 pandemic as a means of continuing patient treatment [71,72]. In a multicentre study in Oman, social support and strong relationships between patients and physicians were found to be important associated factors of better medication adherence in patients with chronic diseases [20]. Oman has also implemented community-based programs, according to which healthcare teams regularly visit remote locations to check on diabetic patients and provide counselling services to them, reducing barriers to access and strengthening the social support framework, especially among patients who live with extended families.

A recent clinical study conducted at a defence hospital clinic in Bahrain examined the impact of extended and intensive patient counselling on the outcomes of diabetes. Patients who underwent a 15 min counselling intervention covering medication adherence, nutrition, and exercise demonstrated a statistically significant decrease in glycated haemoglobin (HbA1c) compared with a six-month follow-up, whereas patients undergoing conventional short consultations showed an increase in HbA1c [73]. These findings suggest potential benefits of patient counselling for glycaemic control; however, improvements in medication adherence cannot be inferred without a direct adherence measure. Kuwait has acknowledged the problem of adherence and has initiated population health campaigns and symposia that focus on empowering patients.

A summary of evidence on associated factors and interventions affecting medication adherence in type 2 diabetes patients across Gulf countries (January 2020–July 2026) is presented in Supplementary Table S4.

## 4. Discussion

### 4.1. Adherence Levels and Measurement Variability

Across the GCC countries, tools used to assess adherence commonly fall within the low-to-moderate range, indicating shortfalls where samples are enriched for uncontrolled disease (e.g., Qatar PHC) [74]. However, differences in measurement instruments (MMAS-8 vs. GMAS vs. MARS), cut-points, and case mix (insulin use, polypharmacy, and glycaemic control) explain much of the variation across the studies [27,42,75]. Nonetheless, several factors were associated with medication adherence, and insulin treatment, increased dosing schedule, increased medication burden, and poor glycaemic control were frequently reported among patients with lower adherence, whereas older age, positive social/family support, and effective patient–clinician relationships were not. Poor glycaemic control was also reported among patients with lower adherence in some studies; however, HbA1c was considered a clinical outcome or associated clinical indicator rather than a direct determinant of adherence. Several recent GCC studies reported high adherence in fewer than half of their participants; however, these findings are study-specific and cannot be generalised as a regional prevalence estimate. Enhancement of adherence in the area demands complex regimen targeting (particularly insulin), incorporation of reminder/monitoring assistance, and the use of family and clinician relationships that are culturally relevant in GCC care models [65].

These findings are generally in line with the rest of the world’s evidence, with a recent systematic review stating that only approximately 54 percent of patients worldwide adhere to oral antidiabetic drugs [9]. Similarly, a recent Western European study reported basal-insulin adherence of 41–64%, with a pooled adherence rate of 55.6%, indicating that suboptimal adherence is not unique to GCC populations [76]. Other Middle Eastern nations, including Jordan and Egypt, also report varying adherence rates [77,78,79]. South Asian data also indicate that about 40% of patients belonged to the poor adherent category [80]. Research in the United States depicts a 65.2% adherence rate [81]. Combined, the literature at hand indicates that, although the problem of adherence is a global occurrence, the available GCC evidence suggests continuing adherence challenges, but international comparisons should be interpreted cautiously because of methodological and measurement differences.

### 4.2. Patient-Related and Sociocultural Factors

The findings also indicate that adherence is shaped by demographic, psychological, social, and cultural factors that should be interpreted within the GCC context. The findings underscore the need to implement health literacy interventions tailored to the cultural and socioeconomic contexts of the GCC. Similar observations have been reported among patients with other non-communicable diseases, such as hypertension, where greater disease knowledge and awareness were positively associated with improved medication adherence [82,83]. These results suggest that the enhancement of patient knowledge and education may positively affect adherence practices in non-communicable conditions because the same tendency was found in the case of hypertension-related medication adherence, which implies that combined educational approaches to diabetes and hypertension may result in a better overall outcome in managing chronic diseases.

In comparison to GCC studies, studies from other countries showed several variations [84,85]. Studies in South Asia and the Middle East also demonstrated that older people are more likely to adhere to chronic disease drugs, whereas gender and marital status were less consistently associated with adherence. In one instance, a 2025 Pakistan study described much higher adherence among patients aged 40 years or older than among patients aged <30 years, and no significant correlation of adherence with gender or marital status [86]. Also, in an Indian study of diabetes (2025), it was found that the only sociodemographic factor associated with adherence was age, and marital status and education did not affect adherence [87]. The same trends are continued in a recent study (2025) conducted in Sudan [88]. Their study indicated that better adherence was associated with higher levels of knowledge and older age, but not with gender or education [88]. Lastly, a 2023 Indian study of 400 T2DM patients found that good disease knowledge was associated with twice the odds of adherence (OR = 1.8), with older age and longer illness duration also found to be associated with adherence [89]. On the whole, all of these post-2020 studies indicate that creating more patient-specific education and adjusting it to demographic subgroups (especially younger patients) would potentially increase adherence to chronic non-communicable diseases globally. Demographic factors should therefore be interpreted as contextual markers rather than direct causal determinants of adherence.

A recent study from Saudi Arabia by Alharbi et al. reported that low adherence is strongly linked to the perception of a more severe illness and vice versa with the patients with higher medication adherence [41]. Other countries’ studies have indicated that personal factors and perceptions, including beliefs about disease severity and effectiveness of medication, self-efficacy, coping planning, perceptions of family support, and communication with healthcare providers, have been reported to be associated with medication adherence in adults with T2DM [90,91,92]. Comparable findings have been reported in European populations, where medication beliefs, satisfaction with communication, and trust in physicians were associated with adherence among patients with T2DM [93]. Addressing misconceptions, improving self-management confidence, patient–provider communication, and the emotional adjustment of patients could thus help to improve adherence in a wide variety of cultural contexts.

### 4.3. Therapy and Healthcare-System Implications

Globally, several studies have explored therapy-related determinants of adherence. Recent studies have shown that insulin therapy often poses adherence challenges due to regimen complexity and the need for regular monitoring [18,76,94]. This highlights the critical role of family medicine and public health programs in supporting patient education, simplifying insulin administration, and ensuring continuous community-based follow-up to maintain optimal diabetes management. Dwiyatna et al. concluded that dose complexity and duration were important factors decreasing adherence in insulin-treated patients, reflecting GCC results on the burden of therapy [95]. By contrast, Aroda et al. demonstrated that patients already familiar with combination regimens were more likely to persist with basal insulin [96]. This is consistent with European evidence showing that complex insulin regimens can increase treatment burden and adversely affect adherence, supporting treatment simplification where clinically appropriate [97]. The influence of therapy-related factors may also vary according to clinical context. For example, insulin use or polypharmacy may indicate more advanced disease in some studies, while in others it may reflect closer clinical follow-up and greater patient education. Combined, the articles highlight the general imperative of enacting policies that facilitate simpler regimens, fixed-dose combinations, and systematic insulin education to improve adherence in any healthcare setting. Therapy-related factors appeared more consistently linked with adherence, although these associations may partly reflect disease severity and treatment burden. Healthcare system factors should also be interpreted in relation to local service arrangements, including access to free or subsidized medications, insurance coverage, appointment availability, and continuity of care. These contextual differences may partly explain variation in adherence findings across GCC settings. It is vital to realise such unseen expenses and provide equal access to prescription medicines among expatriates.

### 4.4. Interpretation of Intervention Evidence

The identified intervention studies suggest potential benefits from educational, pharmacist-led, and technology-supported approaches, although the available evidence remains limited and heterogeneous. Patient and pharmacist education, facilitating patient follow-up and applying technology may offer potential benefits for medication adherence and clinical outcomes, although further robust evaluation is needed.

In comparison with the GCC studies, multifaceted interventions, including patient education, behavioural counselling, clinical simplification, digital technologies, and psychosocial support, have been strongly supported in the global arena to improve medication adherence in T2DM patients. A study on mobile-application interventions indicates that there are weak but significant differences in adherence and glycaemic control [98]. However, digital health interventions led by a pharmacist can consistently support adherence and metabolic outcomes [99]. Another study also supports the idea that interventions led by pharmacists have a significant positive effect on HbA1c, blood pressure, and treatment adherence. Additionally, the study of pharmacist-led models of diabetes reveals that the combination of education, medication assessment, counselling, and lifestyle support is characteristic of successful programs [100].

Interventions directly evaluated in the included GCC studies included structured education, pharmacist-led care, follow-up support, telemonitoring, and selected digital tools, whereas broader family-based, system-level, and large-scale digital adherence strategies should be considered promising approaches requiring further evaluation. Intervention evidence was comparatively limited, but education, pharmacist-led care, follow-up support, and digital tools appeared promising across GCC settings. Overall, the intervention evidence should be interpreted cautiously because studies varied in design, sample size, follow-up duration, and outcome measures. Qatar data also showed that MMAS-8 adherence was not significantly correlated with HbA1c in one primary-care cohort, reinforcing the need to distinguish medication-taking behaviour from glycaemic outcomes [101].

## 5. Knowledge Gaps and Future Directions

### 5.1. Knowledge Gaps

The present review identified several knowledge gaps. To begin with, evidence varies by country: Saudi Arabia and the UAE have several recent studies available, but the available evidence in Kuwait and Bahrain is scarce, and in most cases, it is qualitative or on older samples. Second, most studies used self-reported adherence instruments (e.g., MMAS-8, GMAS, or author-developed), which has several limitations, including recall and social desirability bias; objective measures, such as pharmacy refill records, electronic monitoring, or biomarker-based proxies, are rarely used. Third, longitudinal studies are rare, and most studies are cross-sectional, making it difficult to infer causal relationships between associated factors, adherence behaviour, and clinical outcomes. Fourth, there is limited evidence on at-risk subgroups, including seniors, rural populations, migrants, and patients with multimorbidity. In addition, although the cultural and religious factors (e.g., Ramadan fasting and family dynamics) are often mentioned, few studies formally measure their effect on adherence in a formal manner or test culturally tailored interventions. Finally, recent Saudi and Omani validation studies broaden the measurement base but also introduce ASMA and MAUQ constructs that are not directly interchangeable with MMAS, GMAS, ARMS, MARS, or PDC.

### 5.2. Future Directions

To bridge these gaps, future research could prioritize country-specific and multicentre studies in underrepresented GCC nations such as Kuwait and Bahrain to ensure a more comprehensive regional picture. Reliability (self-report) should be further reinforced by incorporating objective adherence measures (e.g., pharmacy refill databases, digital adherence trackers, and smart pillboxes). Longitudinal cohort studies would be useful to assess time-varying adherence, particularly in relation to disease progression, the intensification of therapies, and healthcare reforms. Further intervention studies may help evaluate pharmacist-led interventions, computer-based health systems, and family-based adherence strategies based on GCC cultural settings. In addition, mixed-methods studies must be conducted to explore the qualitative components of patient beliefs, social support systems, and barriers within the system. Lastly, aligning research with the Saudi Vision 2030 health transformation objectives (specifically in Saudi Arabia) presents an opportunity to integrate adherence improvement measures with broader chronic disease management and digital health systems, with both scientific and policy implications.

## 6. Limitations

Being a scoping review, this work possesses a number of limitations. To begin with, the synthesis relies on published peer-reviewed articles found in the chosen databases, and relevant grey literature, local reports, or unpublished data may have been excluded. Second, the majority of the studies included in the review utilized a cross-sectional design, limiting the assessment of temporal relationships. Moreover, the frequent reliance on self-reported adherence measures may have introduced recall and reporting biases. Third, the review was limited to publications written in English, which may have omitted evidence published in Arabic. Furthermore, variation in adherence measurement tools, cut-off definitions, clinical settings, sample characteristics, and populations across GCC countries limits direct comparison across studies, including those conducted within the same country. Finally, consistent with the objectives of a scoping review, no formal risk-of-bias assessment or certainty-of-evidence grading was undertaken; therefore, the findings should be interpreted as a mapping of the available evidence rather than an assessment of intervention effectiveness or evidence quality.

## 7. Conclusions

This scoping review indicates that medication adherence among patients with T2DM is an important challenge in the GCC countries, despite well-developed healthcare systems and generally available medications. Data since 2020 suggest that reported adherence levels varied considerably across individual studies, with variability across countries, populations, and measures. Associated factors are multifactorial and include patient-related, therapy-related, healthcare-system, and sociocultural factors. Interventions such as pharmacist-led care, digital health applications, and culturally adapted educational programs may offer potential benefits, although evidence of their effectiveness remains limited and heterogeneous. To enhance adherence in the GCC, policymakers may consider multilevel, culturally sensitive strategies. Such strategies could include combining patient and family education, streamlining medication regimens, reinforcing provider–patient communication, and capitalizing on digital health solutions. Adherence-improvement initiatives could be aligned with national health transformation agendas, including Saudi Arabia’s Vision 2030, to ensure sustainability and scalability. Finally, longitudinal, multicentre, intervention, and mixed-methods studies would be valuable to strengthen the regional evidence base.

## Figures and Tables

**Table 1:** Overview of search strategies.

Category	Description
Databases searched	PubMed, Scopus, Web of Science, Saudi Digital Library (SDL)
Timeframe	January 2020–July 2026
Keywords/Search terms	Combinations of: ‘type 2 diabetes’, ‘diabetes mellitus’, ‘medication adherence’, ‘drug compliance’, ‘determinants’, ‘predictors’, ‘barriers’, ‘interventions’, AND country names (Saudi Arabia, United Arab Emirates, Kuwait, Oman, Qatar, Bahrain). Boolean operators AND/OR applied.
Inclusion criteria	Studies conducted in GCC countries, involving adult patients with T2DM, that report on medication adherence levels, determinants, or interventions, published in peer-reviewed English-language journals.
Exclusion criteria	Non-peer-reviewed sources (conference abstracts, commentaries, editorials), studies published before 2020, and studies limited to type 1 diabetes, gestational diabetes, or paediatric populations.
Screening process	Titles and abstracts were independently screened by two reviewers; any disagreements were resolved by a third reviewer. Eligible full-texts were retrieved, and reference lists were hand-searched for additional studies. Duplicates across databases were removed before analysis.

**Table 2:** Country-level summary of medication adherence evidence across GCC countries.

GCC Country	Overall Evidence Pattern	Main Associated Factors/Findings	Evidence Gaps/Distinctive Features
Saudi Arabia	Wide variability across settings/tools	Knowledge, treatment burden, age, family support	Largest evidence base; substantial methodological heterogeneity
UAE	Highly variable estimates	Side effects, treatment confidence, digital care	Some very high and very low estimates
Qatar	Context-dependent adherence	Disease control/self-care	Lower adherence in uncontrolled diabetes samples
Oman	Relatively favourable in some PHC studies	Social support, patient–physician relationship, dosing frequency	Limited T2DM-specific evidence
Kuwait	Limited quantitative adherence data	Information-seeking, family/social media influences	Major evidence gap
Bahrain	Moderate/high adherence in available evidence	Depression/psychological factors	Limited number of studies

## Data Availability

No new data were created or analysed in this study. Data sharing is not applicable to this article as it is based on previously published studies available in public databases and scientific literature.

## Supplementary Materials

The following supporting information can be downloaded at https://www.mdpi.com/article/10.3390/healthcare14213716/s1: Supplementary Table S1: Preferred Reporting Items for Systematic reviews and Meta-Analyses extension for Scoping Reviews (PRISMA-ScR) Checklist; Supplementary Table S2: Database-Specific Literature Search Strategies; Table S3: Comparison of medication-adherence measurement approaches reported in GCC type 2 diabetes studies; Table S4: Summary of Evidence on Determinants and Interventions Affecting Medication Adherence in Type 2 Diabetes Patients Across Gulf Countries (January 2020–July 2026).
