# Supplementary material for: Medication Adherence in Adults with Type 2 Diabetes Mellitus Across Gulf Cooperation Council Countries: A Scoping Review of Determinants, Measurement Approaches, and Intervention Evidence
Source: Healthcare (Basel). 2026 Nov 2;14(21):3716. doi: 10.3390/healthcare14213716 (PMC13763341; doi:10.3390/healthcare14213716)
Supplement: Supplementary file 1 [file healthcare-14-03716-s001.zip › Supplementary Table S2 - Database Search Strategies.pdf]

Supplementary Table S2. Database-Specific Literature Search Strategies

Database / platform	Records retrieved	Search string / recorded query	Filters / limits
PubMed	910	("Diabetes Mellitus, Type 2"[MeSH Terms] OR "type 2 diabet*"[Title/Abstract] OR T2DM[Title/Abstract]) AND ("Medication Adherence"[MeSH Terms] OR "medication adheren*"[Title/Abstract] OR "medication complian*"[Title/Abstract] OR "drug adheren*"[Title/Abstract] OR "drug complian*"[Title/Abstract] OR "medication persist*"[Title/Abstract]) AND ("Saudi Arabia"[Title/Abstract] OR "United Arab Emirates"[Title/Abstract] OR UAE[Title/Abstract] OR Oman[Title/Abstract] OR Qatar[Title/Abstract] OR Kuwait[Title/Abstract] OR Bahrain[Title/Abstract] OR GCC[Title/Abstract] OR "Gulf Cooperation Council"[Title/Abstract])	Publication date window: 2020/01/01–2026/07/31; English/age/article-type filters: verify against PubMed History
Scopus	1,012	TITLE-ABS-KEY("type 2 diabet*" OR T2DM) AND TITLE-ABS-KEY("medication adheren*" OR "medication complian*" OR "drug adheren*" OR "drug complian*" OR "medication persist*") AND TITLE-ABS-KEY("Saudi Arabia" OR "United Arab Emirates" OR UAE OR Oman OR Qatar OR Kuwait OR Bahrain OR GCC OR "Gulf Cooperation Council")	PUBYEAR > 2019 AND PUBYEAR < 2027; precise July 2026 cutoff, language/document filters: verify against Scopus history
Web of Science Core Collection	782	TS=("type 2 diabet*" OR T2DM) AND TS=("medication adheren*" OR "medication complian*" OR "drug adheren*" OR "drug complian*" OR "medication persist*") AND TS=("Saudi Arabia" OR "United Arab Emirates" OR UAE OR Oman OR Qatar OR Kuwait OR Bahrain OR GCC OR "Gulf Cooperation Council")	Publication years 2020–2026; coverage through 31 July 2026 and database editions/language/document filters: verify against WoS history
Saudi Digital Library (SDL)	753	The search covered type 2 diabetes mellitus, medication adherence, determinants, barriers, interventions, and GCC country names, using combinations of Boolean operators (AND/OR).	Record platform, searched fields, Boolean/wildcard syntax, 1 Jan 2020–31 Jul 2026 date range and filters as actually applied

Publication eligibility: 1 January 2020–31 July 2026 | Final search of all four databases: 7 August 2026
