# Supplementary material for: Medication Adherence in Adults with Type 2 Diabetes Mellitus Across Gulf Cooperation Council Countries: A Scoping Review of Determinants, Measurement Approaches, and Intervention Evidence
Source: Healthcare (Basel). 2026 Nov 2;14(21):3716. doi: 10.3390/healthcare14213716 (PMC13763341; doi:10.3390/healthcare14213716)
Supplement: Supplementary file 1 [file healthcare-14-03716-s001.zip › Supplementary TableS3.pdf]

Supplementary Table S3. Comparison of medication-adherence measurement approaches reported in GCC type 2 diabetes studies

Instrument	Items/domains	Measure type	Scoring / adherence thresholds	Validation and Arabic-language status	GCC country / cited examples	Study population	What is measured
MMAS-8	8 items; medication-taking behaviours	Self-report	Score 0–8; commonly low <6, medium 6–<8, high 8	Original MMAS-8 validated; Arabic/version-specific validation must be checked per study	Saudi Arabia [41]; UAE [19]; Qatar [67]; Oman-containing multicountry sample [76]	Adults with diabetes/T2DM in clinical or community samples	Medication-specific
MMAS-4 / Morisky–Green–Levine	4 yes/no items	Self-report	Score 0–4; category direction and cutoffs require study-specific confirmation	Established measure; verify Arabic version and local validation used	Saudi Arabia [11,59]; Oman [27]	Adults with T2DM; PHC/outpatient samples	Medication-specific
GMAS	11 items; 3 domains (patient behaviour, additional disease/pill burden, cost-related nonadherence)	Self-report	Total score 0–33; adherence bands depend on published GMAS rules and version	Originally developed/validated in regional populations; verify Arabic translation/validation for each application	Saudi Arabia [11,36,39]; Bahrain [55]	Adults with T2DM in clinical/community settings	Medication-specific
MARS-5	5 items; medication-taking behaviours	Self-report	Five Likert items; total commonly 5–25; adherence cutoffs study-dependent	Instrument has validation literature; Arabic/local validation to verify	Saudi Arabia [31,51]	Adults with T2DM, including intervention/outpatient cohorts	Medication-specific
MARS (version unspecified)	Number of items/version not confirmed in extracted table	Self-report	Study-specific scale/scoring; verify original article	Arabic/local validation and scale version not documented in extraction	Saudi Arabia [50]	Adults with diabetes in reported study (verify T2DM-only subgroup)	Medication-specific if standard MARS used; confirm version
ARMS / ARMS-12	12 items; medication-taking and refill behaviours	Self-report	12–48, lower scores generally indicate better adherence; categorical thresholds not universal	Original scale validated; verify Arabic version/cutoff in each GCC study	Saudi Arabia [33,47]	Adults with T2DM	Medication/refill-specific
SDSCA medication domain	Medication items within wider self-care assessment	Self-report	Days per week (0–7), as applicable; not equivalent to global adherence scale	SDSCA has multiple language versions; check version actually used	Discussed in main manuscript; country-level study attribution requires confirmation	Adults with diabetes/T2DM	Medication subdomain within broader self-management
ASMA questionnaire	Four adherence-related domains (reported in included study)	Self-report	Initiation, implementation, persistence; study-specific item scoring and thresholds	Instrument-development/psychometric validation in Saudi T2DM sample [29]; Arabic administration details to verify	Saudi Arabia [29]	145 adults with T2DM receiving oral glucose-lowering medication	Medication-specific
MAUQ-ar-OM	Multiple domains; bifactor model reported (exact item count to confirm)	Self-report	Domain/overall attitudes-related scores; no universal adherence prevalence cutoff established	Arabic translation, cultural adaptation and validation studied in Oman [34]	Oman [34]	300 Arabic-speaking adults with T2DM	Broad medication-adherence attitudes/constructs; not directly interchangeable with MMAS
IADMAS	Item/domain number requires verification from source	Self-report	Original study-defined optimal vs suboptimal adherence categories	Originating instrument and Arabic/local validation require verification	Saudi Arabia [57]	393 middle-aged adults with T2DM	Medication-specific
Modified Morisky Scale (MMS)	Modified Morisky version; items to verify	Self-report	Study-specific scoring/cutoffs	Version and Arabic validation not recorded in extraction	Saudi Arabia [52]	Adults with T2DM in pharmacist-related intervention study	Medication-specific
PDC	Claims/refill-based coverage over observation period	Objective administrative proxy	Covered days ÷ eligible days ×100%; ≥80% often used, verify study threshold	Not a questionnaire; depends on accurate prescribing/dispensing records	Saudi Arabia [28,33]	Adults prescribed glucose-lowering medication	Medication-availability proxy, not ingestion

Instrument	Items/domains	Measure type	Scoring / adherence thresholds	Validation and Arabic-language status	GCC country / cited examples	Study population	What is measured
MPR	Dispensed-day supply relative to observation period	Objective administrative proxy	Days supplied ÷ period days; thresholds and handling of overlaps vary	Not linguistic/psychometric; depends on refill record completeness	UAE [71]	Adults receiving glucose-lowering treatment	Medication-availability proxy, not ingestion
Dosing diary / app engagement	Diary entries, dose-instruction adherence or app interactions; variable metrics	Patient report / digital-use proxy	Study-specific: e.g. proportion of days dosing instructions followed; app use alone ≠ adherence	No common validated cutoff; methodology differs by study	Saudi Arabia/UAE/Kuwait [56]; UAE [70]; Saudi Arabia/Kuwait [58]	Adults with T2DM in digital-care or Ramadan studies	Dosing behaviour when directly assessed; app use/clinical change not adherence

Notes: MMAS = Morisky Medication Adherence Scale; GMAS = General Medication Adherence Scale; MARS = Medication Adherence Report/Rating Scale (the precise version must be checked); ARMS = Adherence to Refills and Medications Scale; SDSCA = Summary of Diabetes Self-Care Activities; ASMA = instrument reported by Aloudah; MAUQ = Medication Adherence Universal Questionnaire; IADMAS = Iraq Anti-Diabetic Medication Adherence Scale; PDC = proportion of days covered; MPR = medication possession ratio.
