# Supplementary material for: Medication Adherence in Adults with Type 2 Diabetes Mellitus Across Gulf Cooperation Council Countries: A Scoping Review of Determinants, Measurement Approaches, and Intervention Evidence
Source: Healthcare (Basel). 2026 Nov 2;14(21):3716. doi: 10.3390/healthcare14213716 (PMC13763341; doi:10.3390/healthcare14213716)
Supplement: Supplementary file 1 [file healthcare-14-03716-s001.zip › Supplementary TableS4 Final and Revised.pdf]

Supplementary Table S4. Summary of Evidence on Determinants and Interventions Affecting Medication Adherence in Type 2 Diabetes Patients Across Gulf Countries (January 2020 – July 2026)

Author (s) and year of publication	Study design	Country	Sample characteristics	Methods	Measurement tool	Key Findings / Interpretations
Alnajjar et al. (2025) [49]	Cross-sectional study	Saudi Arabia	n=732 adults with T2DM attending government PHCs in Al-Ahsa, Saudi Arabia.	Questionnaire-based cross-sectional assessment of adults with T2DM in Saudi Arabia; evaluated refill/medication adherence and self-reported reasons for non-adherence using stratified random sampling.	Adherence to Refills and Medications Scale (ARMS)	Medication adherence was seen in 52.5% of type 2 diabetes mellitus patients. 47.5% of patients were non-adherent. Reasons for non-adherence to medication were forgetfulness, experiencing/fear of side effects.
Zakaria et al. (2024) [69]	Retrospective case-control observational study	United Arab Emirates	n=262 adults with T2DM (162 hybrid-care; 100 physical-only); 76.7% male.	Patients were divided into a case group and a control group<br>The case group is a hybrid continuous care group that had a minimum of 2 in-clinic visits and follow-up once every 2 weeks with a physician/coach via chat function on the app. A minimum of 1 reading of body weight is received on the app for every 30 days.<br>The control group had a minimum of 2 in-clinic visits without any virtual engagement on the app. It is an episodic, physical-only care group.	Digital engagement data and clinical/metabolic outcomes; no formal medication-adherence scale	The hybrid-care group had a mean HbA1c reduction of 2.19% over 90 days, with greater engagement associated with larger reductions. Participants with at least 11 interactions had a 2.38% HbA1c reduction and a 6.0 kg reduction in weight.

Author (s) and year of publication	Study design	Country	Sample characteristics	Methods	Measurement tool	Key Findings / Interpretations
Meer et al. (2024) [56]	Qualitative study	Kuwait	n=27 adults with T2DM recruited across healthcare settings in Kuwait.	The grounded theory method was used. A systematic qualitative approach with inductive and iterative data collection was used. Purposive convenience sampling techniques were used.	Qualitative interviews; no formal adherence scale.	Throughout the transitional phases, participants' information behavior was dynamic and ever-changing based on their shifting information demands.<br>Patients had a range of dynamic emotions during the acceptance phase of the diabetes diagnosis, progressing from denial to acceptance.<br>Medication adherence and self-care management were affected in patients who were in the denial stage of Diabetes Mellitus
Khardali et al. (2024) [62]	Qualitative study	Saudi Arabia	n=15 patients with uncontrolled T2DM; mean age 46.3 years; 60% male.	A qualitative research study with face-to-face interviews was conducted, facilitating an in-depth exploration of patients' views.<br><br>A Convenient purposive sampling technique was used	Qualitative interviews; no formal adherence scale.	Medication adherence was influenced by knowledge and beliefs, polypharmacy and medication-related problems, patient-provider relationships, financial constraints, social stigma, and family support. Mobile health applications were suggested as a potential facilitator.

Author (s) and year of publication	Study design	Country	Sample characteristics	Methods	Measurement tool	Key Findings / Interpretations
Albabtain et al. (2024) [53]	Randomized control trial	Saudi Arabia	n=160 patients with uncontrolled diabetes; mean age 50 years; 68.1% male.	Six-month, two-arm, open-label community pharmacy-based medication therapy management intervention with qualitative interviews; compared pharmacist-led care with standard care among patients with uncontrolled diabetes.	Medication Adherence Report Scale (MARS-5); Diabetes Distress Scale (DDS); Patient Satisfaction with Pharmacist Services Questionnaire (PSPSQ 2.0)	Between-group HbA1c differences were 0.02% at 3 months and 0.2% at 6 months and were not statistically significant. At 6 months, participants receiving medication therapy management had higher adherence (76.9% vs 29.7%; OR 7.89) and greater satisfaction than those receiving standard care.
Hasan and Musameh (2020) [73]	A Prospective Study	Bahrain	n=169 adults with T2DM (83 intervention; 86 control), aged >=35 years.	Patients with diabetes mellitus are divided into a control group and an intervention group.<br>The control group had 7 7-minute education sessions, and the intervention group had 15 15-minute education sessions.<br>HbA1c levels were measured before the session and after 6 months.	HbA1c/clinical outcome assessment; formal adherence scale not specified.	A statistical reduction in HbA1c level was seen in the intervention group, which was attributed to the detailed counselling for patients on diet and medications.<br>An increase in HbA1c levels was seen in the control group patients.

Author (s) and year of publication	Study design	Country	Sample characteristics	Methods	Measurement tool	Key Findings / Interpretations
Alharbi et al. (2024) [41]	Descriptive correlation study	Saudi Arabia	n=365 adults with T2DM; mean age 50.9 years; 53.4% female.	Correlational assessment of adult patients with T2DM in Saudi Arabia; examined the relationship between illness perception domains and medication adherence using a convenience sample.	Brief Illness Perception Questionnaire (B-IPQ); 8-item Morisky Medication Adherence Scale (MMAS-8)	Medication adherence was moderate. Lower adherence was associated with perceiving more severe consequences, stronger illness identity, and greater emotional responses; higher adherence was associated with personal control, belief in treatment effectiveness, and better illness understanding.
Khardali et al. (2024) [40]	Cross-sectional study	Saudi Arabia	n=309 adults with T2DM in Jazan, Saudi Arabia.	Single-centre cross-sectional study in Jazan, Saudi Arabia; collected self-administered questionnaire data and reviewed medical records to assess adherence and associated demographic/clinical factors.	Two different approaches were used for data collection.<br>First approach: Self-administered questionnaire & General Medication Adherence Scale (GMAS)<br><br>Second approach: review of patient medical records.	Most of the study participants (66%) were adherent to the anti-diabetic medication.<br><br>Medication adherence had a significant association with patients' age (>50 years), being married, residing in urban areas, and having a glycated hemoglobin of >8%.<br><br>Patients without co-morbidities were more adherent to anti-diabetic medication.

Author (s) and year of publication	Study design	Country	Sample characteristics	Methods	Measurement tool	Key Findings / Interpretations
Alsaidan et al. (2023) [42]	Multi-site cross-sectional study	Saudi Arabia	n=390 adults with T2DM (>=18 years) attending 20 PHCs in Eastern Province.	The consecutive sampling method was used.<br><br>Out of 36 primary health centers (PHCs) located in the Eastern Province of the Kingdom of Saudi Arabia, a random selection of 20 PHCs was selected.<br><br>Every 5th type 2 Diabetes Mellitus patient was selected for the study	The authors made self-administered adherence scale	A statistically significant association was found between female gender and low medication adherence.<br><br>Lower odds of low medication adherence are commonly seen in people who have had a longer duration of diabetes.<br><br>Positive correlation is seen between medication adherence and illness perception.<br><br>A positive correlation is observed between knowledge of diabetes and medication adherence.
Al Shidhani et al. (2022) [27]	Cross-sectional study	Oman	n=202 adults with T2DM in primary care in Oman.	Primary-care-based cross-sectional assessment of adults with T2DM in Oman; evaluated polypharmacy, medication compliance, and related demographic/clinical characteristics.	The Morisky–Green–Levine score was used to determine medication adherence.	Polypharmacy was reported in 83.1% of participants, while 65% reported high treatment adherence. Polypharmacy was significantly associated with age, comorbidities, and duration of T2DM.

Author (s) and year of publication	Study design	Country	Sample characteristics	Methods	Measurement tool	Key Findings / Interpretations
Alanazi and Alatawi (2022) [43]	Cross-sectional study	Saudi Arabia	n=380 adults with diabetes in Tabuk, Saudi Arabia.	A convenient non-probability sampling method was used.<br><br>The chi-square test was used to compare qualitative data between two groups.	Self-reported diabetes treatment adherence questionnaire	Medication adherence was seen in 77.1% of diabetes mellitus patients. 22.9% of patients were non-adherent to medication.<br>One third of the patients were regular on follow-up and never missed any medication<br>The most common cause of missed medication doses was forgetfulness.
Asheq et al. (2021) [19]	Descriptive, cross-sectional study	United Arab Emirates	n=180 adults with T2DM; 52.8% male and 47.2% female.	Descriptive cross-sectional study among patients with T2DM in the UAE during the COVID-19 pandemic; assessed antidiabetic medication adherence and factors associated with adherence.<br>Convenience sampling was done.	The Morisky Medication Adherence Scale (MMAS) was used for data collection	Low, medium, and high adherence were reported by 61.7%, 28.9%, and 9.4%, respectively. Lower adherence was associated with being unmarried and aged 30-59 years, whereas non-insulin therapy and confidence in taking diabetes medication were associated with higher adherence.

Author (s) and year of publication	Study design	Country	Sample characteristics	Methods	Measurement tool	Key Findings / Interpretations
Alsunni et al. (2020) [63]	Descriptive, Cross-sectional study	Saudi Arabia	n=107 adults with T2DM; mean age 52.9 years; 63.6% male.	Purposive sampling was done<br><br>ANOVA test reflected significant difference of attitude score due to education<br><br>Tukey's HSD test (post hoc) was used to identify the groups showing significant differences.	Knowledge, attitude and practice (KAP) questionnaire on Ramadan fasting in T2DM	Males with education status more than secondary school had shown positive association with attitude.<br><br>During fasting for Ramadan, 32% adjusted the timing of medication without doctor's advice. The dosage or frequency of using the medication was done by 28% of the patients without doctor's advice.<br><br>Lifestyle modifications through Ramadan focused education can help in reducing the hypoglycemic events and achieving proper metabolic control.
Ibrahim et al. (2021) [75]	Cross sectional study	Two countries that includes Oman	Adults with chronic diseases from Oman and the USA; Omani survey included 776 respondents.	Comparative cross-sectional study of patients with chronic disease from Oman and the United States; examined medication experiences, beliefs, and low medication.<br><br>Convenience sampling was done.	The Morisky Medication Adherence Scale (MMAS-8) was used to assess medication adherence.	Medication adherence was low(MMAS – 8 score < 6) in 56% of Omani and 52% of U.S groups<br><br>People who had positive experience and beliefs with medication showed better medication adherence.

Author (s) and year of publication	Study design	Country	Sample characteristics	Methods	Measurement tool	Key Findings / Interpretations
Al-Noumani et al. (2023) [20]	Cross-sectional study	Oman	n=800 Omani adults with chronic diseases; 51.9% female; 74% aged >=46 years.	Multicenter cross-sectional study among Omani patients with chronic diseases; evaluated health literacy, perceived social support, patient-doctor relationship, and medication adherence.<br><br>Convenience sampling method was used	The Brief Health Literacy Screening Tool, The Multidimensional Scale of Perceived Social Support (MSPSS), A Patient-Doctor Relationship Questionnaire (PDRQ-9), The Adherence in Chronic Disease Scale (ACDS) were used to measure study variables	Medication adherence was positively associated with perceived social support and patient-physician relationship<br><br>Higher medication adherence was seen in patients who were unemployed, had a better patient-physician relationship and also who had great social support.<br><br>Lower medication adherence was seen in patients who had disease duration of greater than 10 years and also who had to take 2-3 times medication / day
Khan et al. (2022) [54]	Multi-arm pre-post prospective study	Saudi Arabia	n=109 adults with T2DM completing follow-up (55 control; 54 intervention); mean age 58.3 years.	Convenience sampling was done. Pharmacist based diabetic intervention (PDIM) was done.<br><br>Pre- and post-analysis was done in a period of 6 months by dividing the participants into control and intervention groups.	Modified Morisky Scale (MMS) was used for assessing the medication adherence	Medication adherence and HbA1c showed significant improvement in the intervention group in the follow-up evaluation after 6 months compared to the control group<br><br>Significant improvement was also seen in the self-care practices of diabetes in intervention group compared to control group

Author (s) and year of publication	Study design	Country	Sample characteristics	Methods	Measurement tool	Key Findings / Interpretations
Farooqi et al. (2022) [68]	Prospective, single center, interventional trial study	United Arab Emirates	Previously lost-to-follow-up adults with T2DM enrolled in a single-centre telemonitoring study.	Simple random sampling technique was used. Single-center interventional telemonitoring study in previously lost-to-follow-up patients with T2DM in the UAE; used remote monitoring and follow-up to assess glycemic and metabolic outcomes.	Telemonitoring data and clinical/metabolic outcomes	HbA1c has reduced from 10.3±1.9% at baseline to 7.4±1.5% at the end of follow up with a mean difference of - 2.9%<br><br>HbA1c was < 7% in 50% of patients after 3 months.<br><br>Telemonitoring showed significant improvement in control of diabetes and also in management of body weight
Shaikh et al. (2023) [22]	Cross sectional study	United Arab Emirates	n=204 adults with T2DM in Ajman, UAE.	Cross-sectional study among patients with T2DM in Ajman, UAE; assessed antidiabetic medication adherence and reported reasons for non-adherence.	Morisky's medication adherence scale was used to determine the adherence	Medication adherence of 91% and 90 % was seen among males and females respectively.<br><br>Nonadherence to medication was seen among patients who had forgetfulness and who experienced unpleasant side effects, had longer duration of diabetes and also who were on multiple medications
Albadrani et al. (2025) [11]	Mixed-method cross-sectional study	Saudi Arabia	Diabetic patients attending PHCs in Madinah; both type 1 and type 2 diabetes were included in the source study.	Mixed-method cross-sectional assessment of cultural, socioeconomic, and clinical determinants.	The Morisky Medication Adherence Scale (MMAS-4) and General Medication Adherence Scale (GMAS) was used	Medication adherence showed significant association between elderly age, higher educational status and also overweight patients with greater than 10 years of diabetes

Author (s) and year of publication	Study design	Country	Sample characteristics	Methods	Measurement tool	Key Findings / Interpretations
AlShayban et al. (2020) [39]	Cross-sectional study	Saudi Arabia	n=318 adult outpatients with T2DM; mean age 44 years; 67.9% male.	Outpatient cross-sectional study among patients with T2DM in Khobar, Saudi Arabia; assessed diabetes knowledge and its association with medication adherence using questionnaire-based data.<br>Convenience sampling was done.	Michigan Diabetes Knowledge Test and General Medication Adherence Scale was used	High adherence to medication was seen in 33% of patients. Half of the patients showed a disease knowledge of 51% to 75%.<br><br>Medication adherence was found to be significant in patients who scored >50% in diabetes knowledge test questionnaire (AOR 4.46, $p < 0.01$)
Munsour et al. (2020) [67]	Randomized control intervention study	Qatar	n=140 adults with T2DM (66 intervention; 74 usual care).	Randomized intervention evaluating tailored consumer medicine information among patients with T2DM.	Morisky Medication Adherence Scale (MMAS-8) was used	The intervention group has shown improved median MMAS-8 scores in the intervention group compared to baseline group in 6 month follow up period and also statistical significance in scores were observed
Mirghani et al. (2023) [52]	Cross-sectional study	Saudi Arabia	n=384 adults with diabetes; 52.3% male; 35% aged 31-50 years; 64.1% had T2DM.	Convenience sampling was used to assess adherence during Ramadan.	Medication Adherence Rating Scale (MARS) was used to assess compliance	Patients having higher HbA1c levels exhibited increased compliance to medication<br><br>Compliance was adversely correlated with ages between 31 and 50 ($\beta = -1.06$, $p = 0.0002$) and positively correlated with ages between 51 and 65 ($\beta = 1.00$, $p = 0.0001$).

Author (s) and year of publication	Study design	Country	Sample characteristics	Methods	Measurement tool	Key Findings / Interpretations
Alshayban et al. (2023) [36]	Cross-sectional study	Saudi Arabia	n=376 adults with T2DM recruited from two hospitals in Eastern Province.	Multicentre cross-sectional study in the Eastern Province of Saudi Arabia; used systematic sampling to assess diabetes medication adherence and associated demographic/clinical factors.	General Medication Adherence Scale (GMAS)was used for assessment of medication adherence	High levels of medication adherence was seen in 42% of patients.<br><br>Low medication adherence was observed in 37% of the patients. Patients with a random blood sugar of >200mg/dl showed lower medication adherence.
Aloudah (2026) [29]	Cross-sectional instrument-development and validation study	Saudi Arabia	n=145 adults receiving oral hypoglycaemic therapy in Riyadh.	Tertiary diabetes centre study in Riyadh among 145 adults receiving oral hypoglycaemic therapy; items were derived from patient interviews and mapped to the Theoretical Domains Framework.	ASMA Medication Adherence Questionnaire; three self-reported ABC taxonomy items	Reported adherence was 97.9% for initiation, 91.8% for implementation, and 84.2% for persistence. The final questionnaire contained four adherence-related domains.
Asrawi et al. (2026) [34]	Cross-sectional psychometric validation study	Oman	n=300 Arabic-speaking adults with T2DM at Sultan Qaboos University Hospital.	Validation study among 300 Arabic-speaking adults with T2DM at Sultan Qaboos University Hospital; included translation, cultural adaptation, and confirmatory factor analysis.	Arabic Medication Adherence Universal Questionnaire for Oman (MAUQ-ar-OM); HbA1c	The bifactor model showed the better fit. Positive attitudes toward healthcare and medication had a small inverse association with HbA1c; other domains and the total score were not significantly associated.
Abudawood et al. (2026) [48]	Analytical cross-sectional study	Saudi Arabia	n=393 adults aged 40-65 years attending King Abdulaziz University Hospital, Jeddah.	Face-to-face survey of 393 adults aged 40-65 years attending King Abdulaziz University Hospital, Jeddah; participants were recruited consecutively.	Iraq Anti-Diabetic Medication Adherence Scale (IADMAS)	Suboptimal adherence was reported by 78.4%, while 21.6% had optimal adherence. Unclear treatment plans, limited explanation of complications, missed follow-up or dispensing dates, and self-discontinuation were associated factors.

Author (s) and year of publication	Study design	Country	Sample characteristics	Methods	Measurement tool	Key Findings / Interpretations
Ali et al. (2025) [28]	Retrospective observational study	Saudi Arabia	n=5,087 adults prescribed semaglutide or empagliflozin.	Electronic-record comparison of 5,087 adults prescribed semaglutide or empagliflozin; adherence and persistence were assessed from medication coverage and treatment gaps.	Proportion of days covered (PDC); persistence based on treatment gaps	PDC-defined adherence was 57.7% for semaglutide and 60.0% for empagliflozin. Persistence was 40% and 45%, respectively; adherence was associated with better glycaemic control.
Amirthalingam et al. (2025) [33]	Cross-sectional study	Saudi Arabia	n=434 adults receiving once-weekly semaglutide in Tabuk.	Outpatient pharmacy study of 434 adults receiving once-weekly semaglutide in Tabuk; self-reported adherence was compared with refill coverage and clinical outcomes.	Adherence to Refills and Medications Scale (ARMS); proportion of days covered (PDC)	Only 32.5% were adherent according to ARMS. Non-adherence was associated with higher BMI and HbA1c, while combined ARMS and PDC adherence was associated with better clinical outcomes.
Hassanein et al. (2025) [58]	Prospective non-interventional study	Saudi Arabia, United Arab Emirates, and Kuwait	n=257 adults with T2DM in the full analysis set; mean age 52.9 years; 58.0% male.	Twenty-week real-world study of adults using oral semaglutide while fasting during Ramadan; administration practices and clinical outcomes were recorded prospectively.	Patient diaries assessing adherence to oral semaglutide dosing instructions	Among participants with diary data, 68.4% followed dosing instructions on at least 80% of recorded days. Adherent participants had greater reductions in HbA1c and body weight.
Al-Sofiani et al. (2025) [59]	Retrospective observational cohort study	Saudi Arabia and Kuwait	n=494 users of the My Dose Coach application receiving clinician-guided basal-insulin titration.	Analysis of data from 494 users of the My Dose Coach application for clinician-guided basal-insulin titration; outcomes were compared by application-use frequency.	My Dose Coach application-use data; fasting blood glucose and HbA1c	Individualised fasting-glucose targets were achieved by 55.9% of users. More frequent application use was associated with faster and better target achievement.

Author (s) and year of publication	Study design	Country	Sample characteristics	Methods	Measurement tool	Key Findings / Interpretations
Alkathiri et al. (2025) [47]	Cross-sectional study	Saudi Arabia	n=327 adults receiving health-coach follow-up across 24 PHCs in Makkah.	Structured interviews with 327 adults receiving health-coach follow-up across 24 primary healthcare centres in Makkah.	Morisky, Green and Levine Medication Adherence Scale (MMAS-4)	High, intermediate, and low adherence were reported by 48.3%, 39.5%, and 12.2%, respectively. Poor family support, difficulty managing diabetes, and medication side effects were associated with non-adherence.
Alubaidi et al. (2024) [57]	Cross-sectional study	Bahrain	n=455 adults with T2DM attending primary care in Bahrain.	Primary-care survey of 455 adults with T2DM assessing depressive symptoms and medication adherence.	Patient Health Questionnaire-9 (PHQ-9); General Medication Adherence Scale (GMAS)	Medication adherence was 79.1%, and depressive symptoms were present in 30.5%. Depressive symptoms were significantly associated with medication adherence.
Almalki et al. (2024) [31]	Cross-sectional study	Saudi Arabia	n=583 adults with T2DM attending PHCs in Riyadh.	Survey of 583 adults with T2DM attending primary healthcare centres in Riyadh; outcomes were compared between telemedicine users and patients receiving in-person care.	Medication Adherence Report Scale (MARS-5); HbA1c and clinical records	Telemedicine users had higher adherence than patients receiving only in-person care (72.9% vs 51.6%); telemedicine use was associated with adherence after adjustment.
El-Deyarbi et al. (2024) [70]	Randomized controlled trial	United Arab Emirates	n=192 adults with T2DM in a 12-month randomized controlled trial.	Twelve-month trial among 192 adults with T2DM comparing usual care with pharmacist-led counselling, medication therapy management, a booklet, and a mobile application.	Medication possession ratio (MPR); medication-adherence questionnaire	Mean total MPR was higher in the intervention group than in controls (0.95 vs 0.92). High self-reported adherence at study exit was also more frequent with the intervention (70.2% vs 48.9%).
